# Molecular Mechanisms of Transcription Initiation at *gal* Promoters and their Multi-Level Regulation by GalR, CRP and DNA Loop

**DOI:** 10.3390/biom5042782

**Published:** 2015-10-16

**Authors:** Dale E.A. Lewis, Sankar Adhya

**Affiliations:** Laboratory of Molecular Biology, National Cancer Institute, National Institutes of Health, Bethesda, MD 20892-4255, USA; E-Mail: adhyas@mail.nih.gov

**Keywords:** activation, repression, DNA looping, transcription, galactose operon

## Abstract

Studying the regulation of transcription of the *gal* operon that encodes the amphibolic pathway of d-galactose metabolism in *Escherichia coli* discerned a plethora of principles that operate in prokaryotic gene regulatory processes. In this chapter, we have reviewed some of the more recent findings in *gal* that continues to reveal unexpected but important mechanistic details. Since the operon is transcribed from two overlapping promoters, *P1* and *P2*, regulated by common regulatory factors, each genetic or biochemical experiment allowed simultaneous discernment of two promoters. Recent studies range from genetic, biochemical through biophysical experiments providing explanations at physiological, mechanistic and single molecule levels. The salient observations highlighted here are: the axiom of determining transcription start points, discovery of a new promoter element different from the known ones that influences promoter strength, occurrence of an intrinsic DNA sequence element that overrides the transcription elongation pause created by a DNA-bound protein roadblock, first observation of a DNA loop and determination its trajectory, and piggybacking proteins and delivering to their DNA target.

## 1. Introduction

The study of the galactose (*gal*) operon, which encodes enzymes for an amphibolic pathway of d-galactose metabolism, first revealed a plethora of gene regulatory mechanisms by which bacterial genes are regulated: (i) beside substrate induction of a specific catabolic pathway or end-product repression of a specific biosynthetic pathway, accumulation or depletion of metabolic intermediates in the cell globally regulates the expression of a wide variety of genes to compensate for the accumulation or depletion [1]; (ii) use of more than one promoter to regulate an operon [2]; (iii) the mechanism of Rho-mediated premature transcription termination [3]; (iv) gene regulation by a DNA element located within a structural gene [4]; (v) DNA looping to repress gene transcription [4]; (vi) the global gene activator, CRP, can also represses a gene [2]; (vii) demonstration of trajectory of DNA loops [5]; and (viii) phage protein mediated transcription anti-termination in bacterial genes [6]. Here we review more recent revelations that provide several new aspects of the multiple regulatory pathways by which *gal* promoters are regulated at the transcription level.

## 2. The *Gal* Operon

**Figure 1:**
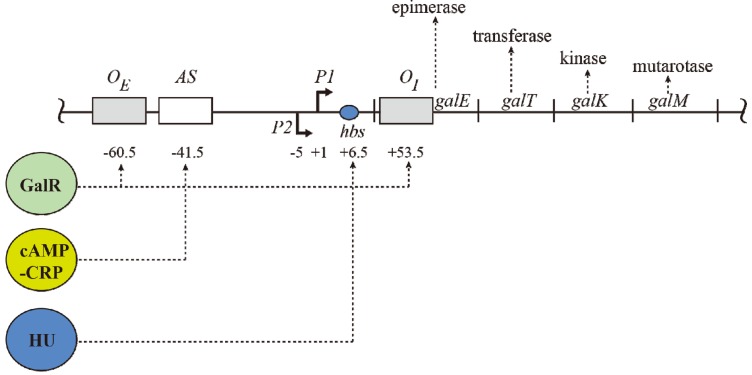
The *gal* operon. The *gal* structural genes, *gal*ETKM, encode the enzymes epimerase, transferase, kinase and mutarotase, respectively. The transcription start point (*tsp*) of *P1* is +1 and that of *P2* is −5 The numbering system is relative to +1, with numbers downstream of +1 as positive (+) and numbers upstream as negative (−). Operators (*O_E_* & *O_I_*), *hbs*: HU binding site, AS: activating site. GalR (green) binds to two operators (*O_E_* & *O_I_*). HU (blue) binds to the HU binding site (*hbs*) and cAMP-CRP (yellow) complex binds to the activating site (AS). The map is not drawn to scale.

The *gal* operon is transcribed from two overlapping promoters, *P1* and *P2*, with transcription start points marked as +1 and −5, respectively (Figure 1) [2,7,8]. Why two promoters? Each promoter responds to different regulators for coping with physiological needs as enzymes encoded in the *gal* operon are needed for both catabolic and anabolic metabolisms. Both promoters are intrinsically expressed at significant levels. Nonetheless, cAMP and its receptor protein CRP complex (CCC) enhances *P1* but represses *P2*, whereas GalR represses *P1* and enhances *P2* (details below). CCC acts by binding to a single site *AS* and GalR to two operators.

## 3. Intrinsic Strength of Promoters

**Figure 2:**
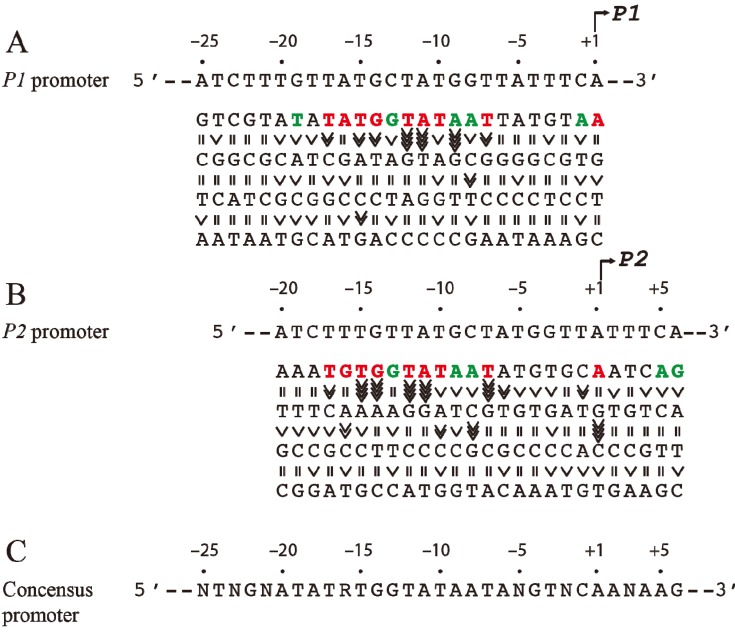
Base pair requirement in *gal* promoters. (**A**) The DNA sequence from −25 to +1 of *P1* and a summary of the effect of base pair changes from +1 to −25 on *P1* transcription; (**B**) The DNA sequence from −20 to +6 of *P2* and a summary of the effect of base pair changes from −20 to +6 on *P2* transcription; (**C**) Consensus promoter region of *P1* and *P2* derived from the results shown in (A) and (B). R = A or G, N = any nucleotide. Base pair is in red if it is unique for promoter function, green if it improves promoter function, and black if it is degenerate. The symbol “>>>>” in vertical shapes represents 4.1-fold or more difference in promoter function from the wild type; “>>>”, 3.1 to 4-fold; “>>”, 2 to 3-fold; “>” less than 2-fold; “=” indicates equal (reproduced with permission from Elsevier, [14]).

Since the *tsps* for *P1* and *P2* are separated by 5-bp (half of a helical turn on B-DNA), the two promoters are located on opposite faces of the DNA. The intrinsic strength of each promoter depends on the contribution of several critical base pairs in the promoter region [9]. Both *P1* and *P2* do not have functional −35 elements but are composed of ex-10 and −10 sequences as authenticated by mutational studies [10,11]. In the absence of regulatory protein, *P2* is transcribed 3-fold more efficiently than *P1* [12]. The intrinsic strength of a promoter was postulated to be dependent on the presence of and the closeness of DNA sequences of the different elements to their consensus forms so that the frequency of occurrence of a base pair at a given position of the element reflects its relative importance in promoter function [13]. But, the significance of the base pair frequency concept in promoter strength was developed without regard to the context sequence. It is probable that the contribution of a base pair to the promoter strength may depend upon the presence of a specific base pair at another seemingly unrelated position in the promoter. This would not be known by looking for consensus sequences among heterologous promoters. A meaningful approach would be to assess the contribution of a base pair at a given position in the promoter under the context sequence that was kept constant. To study the effects of individual base pairs on the intrinsic strength of the promoters, each base pair in the overlapping *gal* promoter region (from −20 to the +5) was mutated systematically to the other three base pairs and the promoter activities were analyzed by an *in vitro* transcription assay [14]. First, it was observed that purines at the non-template strand at the *tsp* of *P1* and *P2* are favorable for the initiation of transcription while pyrimidines are unfavorable with a preference for A = G >> C = T at the *tsp* (Figure 2). The *tsp* is determined by counting 12 base pairs from the “master base” −11A (see below) located within the −10 element of *P1* and *P2* [15,16]. Next, base pairs −7T, −11A, and −12T were found to be critical determinants of promoter activity. Mutating the corresponding −7T, −11A or −12T to another base inactivated *P1* and *P2* [14]. In addition, base pairs in the ex-10 elements (−15T and −14G) of *P1* and *P2* were also critical for promoter activities as expected from previous results. In summary, the base pair frequency within known consensus elements correlated well with promoter strength. Surprisingly however, *P1* and *P2* promoter strengths increased by substitution of several native base pairs by some others located in the −20 to −16 segment, *i.e.*, outside the ex-10 and −10 standard elements of both promoters with a consensus sequence of ^−20^ATATA/G^−16^ for the region; no sequence requirement in that segment was predicted before. How this new sequence element influences promoters is unknown. The results of the exhaustive mutational analysis about DNA sequence requirements in *gal* promoters are summarized in Figure 2 [14].

The steps of closed and open complex formation in *gal* promoters were studied by the indirect abortive initiation method [17]. The mechanism of base pair opening during transcription initiation by RNA polymerase at the *galP1* promoter was directly assayed by 2-aminopurine (2,AP) fluorescence [18]. The fluorescence of 2,AP is quenched when present in DNA duplex and enhanced when the 2,AP:T base pair is distorted or deformed. The increase of 2,AP fluorescence was used to monitor base pair distortion at several individual positions in the promoter. Base pair distortions during isomerization were observed at every position tested except at −11 in which the substitution created a defective promoter. The isomerization appeared to be a multi-step process. Three distinct hitherto unresolved steps in kinetic terms were observed, where significant fluorescence change occurred: a fast step with a half-life of around 1 s, which is followed by two slower steps occurring with a half-life in the range of minutes at 25 °C. Contrary to commonly held expectations, base pairs at different positions opened by 2,AP assays without any obvious pattern, suggesting that base pair opening is an asynchronous multi-step process. Note that 2,AP was used only at positions where there was an A in the “opening” region of the promoter.

The −11A within the −10 box is termed the “master control switch” because DNA melting and DNA strand opening first occur at −11 during isomerization from the closed to the open complex followed by opening at subsequent positions (−11 to +3) [15,19,20]. A mutant −11A does not allow base pairs at other positions to open whereas the reverse is not the case. Crystal structure studies showed that −7T and 11A flip out into hydrophobic pockets in an open complex [21,22]. This explains why any mutation in −7 and −11 positions results in the loss of *gal* promoter activities [14,15,19]. It also explains why −7T and −11A bases are highly conserved in the −10 element of promoters and play important roles during the formation of the open complex. It has been proposed that during isomerization, strand opening occurs from −11 to +3 to form a single-stranded DNA bubble, while −12T remains as part of the upstream double-stranded DNA bound to RNAP [14].

## 4. Role of CCC

**Figure 3:**
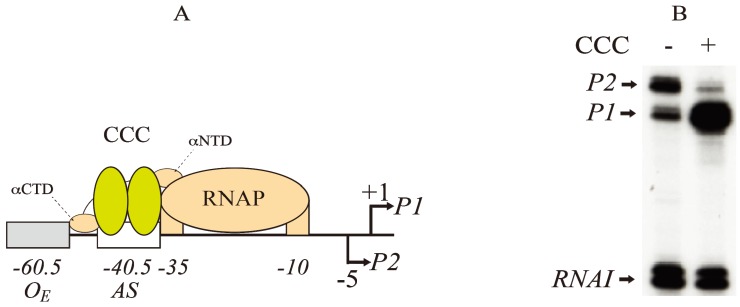
Regulation of *gal* promoters by CRP complex (CCC). (**A**) Model of interactions between CCC (yellow) at the activating site (−40.5) and RNAP (brown) at the −10 and −35 elements of *P1* (+1). *P2* is located at −5 The αNTD and αCTD of RNAP contact both subunits of Class II promoters at CCC as shown in Figure 4 (adapted from [25]); (**B**) RNAs made typically from *P1* and *P2* promoters in the absence (−) and presence (+) of CCC as analyzed by gel electrophoresis. The concentrations of cAMP and CRP are 100 μM and 50 nM, respectively. *RNAI* is a control RNA in the plasmid (reproduced with permission from Elsevier [14]).

The *gal* operon includes a 16-bp activating site (*AS*) located at −40.5 that binds the regulator CCC for activating *P1* and repressing *P2* (Figure 3A) [8,23,24,25]. A typical result of CCC action at the *gal* promoters is shown in Figure 3B. The overlapping of the *AS* at −40.5 with the −35 element of *P1* is a feature of CCC-regulated Class II promoters. In contrast, in Class I promoters, the *AS* is located upstream (−61.5) to the promoter region for RNA polymerase (RNAP).

CCC represses *P2* by decreasing open complex formation of RNAP. In contrast, CCC activates *P1* by increasing both closed complex formation and isomerization from the closed complex to the open complex of RNAP [17]. The *AS* of CCC is located on the same face as RNAP at *P1* and on the opposite face of RNAP at *P2*. By binding to *AS*, CCC switches transcription initiation from *P2* to *P1*. The activation of *P1* by CCC is also dependent on the superhelical density of the DNA. The maximal *P1* activity (12-fold) was observed at a superhelical density of −0.051, but the activity decreases at both higher and lower densities on a plasmid of 3528 bp [26]. In the absence of CCC, *P2* activity is maximal (2-fold) also at a superhelical density of −0.051.

**Figure 4:**
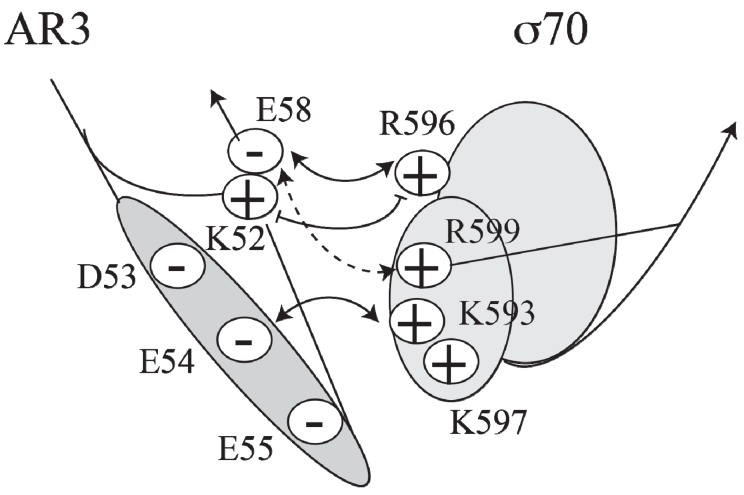
Model of the interactions between AR3 and sigma 70 (adapted from [27]).

## 5. CCC and RNAP Interactions

Cooperative binding between CCC and RNAP was demonstrated using DNase I protection assays [28]. Amino acids involved in the interaction of CCC with RNAP that produced cooperative binding are confined to three activating regions (AR1, AR2 and AR3) of CRP [29,30,31,32,33,34,35,36,37]. The alpha carboxyl-terminal domain (αCTD, residues 249–329) of RNAP interacts with CRP at AR1 (residues 156–164), the alpha amino-terminal domain (αNTD, residues 8–235) interacts with CRP at AR2 (residues 1, 9, 21, 96 and 101), and the σ70 subunit of RNAP interacts with CRP at AR3 (residues 52–58) [25,29,34,38]. The crystal structure of CCC-αCTD-DNA complex has been determined [36].

CCC induces transcription at *P1*, a Class II promoter, by making three different activatory contacts with different surfaces of holo RNA polymerase [34]. One of the contacts is located in the downstream subunit of the CRP dimer at the *AS* site and has been predicted to interact with region 4 of the RNAP σ70 subunit [27,39]. A cluster of negatively charged residues (D53, E54, E55 and E58) in AR3 of CRP interacts with a cluster of positively charged residues (K593, K597, R599 and R596) in σ70 (Figure 4).

RNAP predominantly forms a binary complex at the *P2* promoter in the absence of CCC and a ternary complex at the *P1* promoter in the presence of CCC. Very high concentrations of heparin are able to dissociate CRP from the *P1* ternary complex without changing the properties of the complex. Thus, CCC is not required for the maintenance of the RNAP complex and plays no role in the subsequent steps in *P1* transcription as was true for several other promoters [40], suggesting that interaction between CCC and RNAP is needed only transiently for the activation of transcription.

## 6. CCC Action on Templates with Single bp Deletions

**Figure 5:**
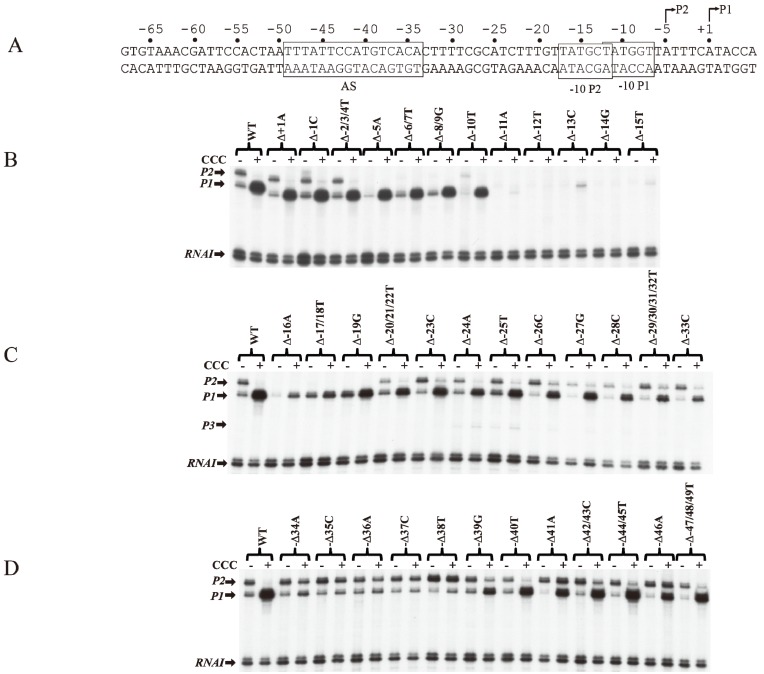
Effect of base pair deletions on *in vitro* transcription of *gal* promoters. (**A**) Sequence of *gal* DNA from −68 to +6 with *tsp* of *P2* (−5) and *P1* (+1). The −10 element of *P1* and −10 element of *P2* are boxed. The CCC site (AS) is also boxed; (**B**) mRNAs made from *P1* and *P2* from WT and mutant templates (Δ−1 to Δ−15) (reproduced with permission from John Wiley and Sons); (**C**) mRNAs made from *P1* and *P2* from WT and mutant templates (Δ−16 to Δ−33); (**D**) mRNAs made from *P1* and *P2* from templates with WT and mutant templates (Δ−34 to Δ−49).

The role of individual base pairs from −49 to +1 on CCC action was investigated by systematically deleting each base pair and monitoring the effect of CCC on *P1* activation and *P2* repression (Figure 5A). Deletion of one base pair from positions +1A to −10T (Δ+1A to Δ−10T) does not affect the activation of *P1* or the repression of *P2* by CCC (Figure 5B). The deletion of 1-bp shifted the next adenine from +3 in WT to +2 in the deletion templates (Δ+1A to Δ−10T), allowing the *tsp* of *P1* to initiate at the new +2A [16]. *P2* with *tsp* of −5A was inactivated with single bp deletion from Δ−5A to Δ−8/9G, because, no adenine or guanine is available from −4 to −2 to initiate *P2*. Single bp deletion of −11A or −12T inactivated both promoters. Interestingly, the *tsp* of *P1* initiated at the new +2A on Δ−11A as observed by the faint transcript band in the presence of CCC. In Δ−12T, *P1* initiates at the WT +1A, suggesting that if the distance from −11T to +1 (12-nt) is shortened, RNAP will choose the next downstream purine to initiate transcription.

The −12T position is the first base of the −10 element of *P1* and the last base of the −10 element of *P2*. It is not surprising that the intrinsic transcription of both *P1* and *P2* was inactivated, and CCC failed to activate *P1*. The −13C to −17T sequence contains the ex-10 of *P1* (^−15^TG^−14^) and the −10 element of *P2* (^−17^TATGCT^−12^). Sigma region 2.5 of RNAP recognizes the ex-10 motif of promoters [41,42,43,44]. Detailed analyses of the ex-10 showed the importance of the ex-10 element in transcription regulation [43]. Deletions of −16A and −17T/−18T result in approximately 5- and 2-fold activation of *P1* by CCC, respectively (Figure 5C). *P2* was inactivated from −5A to −19G because its *tsp*, ex-10 and −10 elements (^−20^TGTTATGCT^−12^) are altered. From −20T to −33C, the regulation of *P1* and *P2* is restored.

CCC is known to protect the *AS* region in the *gal* DNA from −50 to −25 bp by DNase I protection assays [10,31,45,46,47]. *AS* contained a non-consensus (NC) $\frac{5-TTTAT-3}{3-AAATA-5}$ half-site and a consensus (C) $\frac{5-TCACA-3}{3-AGTGT-5}$ half-site separated by a 6-bp spacer [48,49,50,51]. The *AS* extends from −49 to −34. Thirteen mutations each of which inhibits CCC action are located in the consensus half-site from −38 to −34 ($\frac{5-TCACA-3}{3-AGTGT-5}$) proximal to the promoters [23,52]. When −34A is deleted, there is only marginal activation of *P1* and repression of *P2* (Figure 5D). There was no noticeable change in *P1* or *P2* levels in the absence or presence of CCC when a base pair in the consensus half-site is deleted. These results suggest that CCC fails to bind to *AS* when a base pair is deleted in the consensus half-site. The basal level of *P2* was increased by 2-fold in Δ−38T. Perhaps a stronger −35 element of *P2* is created with Δ−38T. The activation of *P1* by CCC was restored with single base pair deletions upstream of the consensus half-site from Δ−39G to Δ−47/48/49T. *P1* was activated only 4-fold in Δ−39G. These results suggest that the 6-bp spacer between the consensus and non-consensus half-sites do not affect CCC binding. These also suggest that mutations of the non-consensus half-site do not affect the activation of *P1* by CCC. Interestingly, Δ−41A and Δ−46A are the only two mutations in *P1,* which were activated 9-fold in Δ−41A and 8-fold in Δ−46A by CCC. However, in both Δ−41A and Δ−46A templates, CCC activated *P2* marginally. Busby and colleagues showed that the consensus half-site is inactivated by three substitution mutations, p35 (−35 CG to GC), p37 (−37CG to AT) and p38 (−38 TA to AT) [23,52]. They also showed that *AS*, unlike in WT, is not protected by CCC in p35, p37 and p38 mutants [10,46]. EMSA shows no stable complexes of CCC binding to a 144-bp DNA fragment containing p35, p37, or p38 mutations [46].

In summary, (i) the distance between −11 and +1 determines the start point selection of *P1* and *P2*. If a purine is not available at +1, RNAP selects the next downstream purine within 12–13 bp from −11A; (ii) the −7T, −11A and −12T are critical bases of the −10 elements of *P1* and *P2* for promoter function. Any deletion or substitution of these bases prevents intrinsic transcription. CCC restores transcription from *P1* in −7T and −12T, but not in −11A; (iii) both base pairs in the ex-10 elements (^−15^TG^−14^) are critical in both *P1* and *P2* because deleting or substituting one of them inactivates both promoters; (iv) any base pair deletion in the spacer region from −20 to −33 does not affect the activation and repression of *P1* and *P2* by CCC, respectively; (v) CCC fails to activate *P1* or repress *P2* when any base pair in the consensus half-site (−34 to −38) of *AS* is deleted; (vi) any base pair deletion except −41 and −46A in the non-consensus half-sites does not affect the regulation of the promoters by CCC. The conclusion from the results of single base pair deletions about the role of base pairs in the promoters are mostly the same as from the results of single base pair substitutions in the *gal* promoters.

## 7. Regulation by GalR-*O_E_* Complex

**Figure 6:**
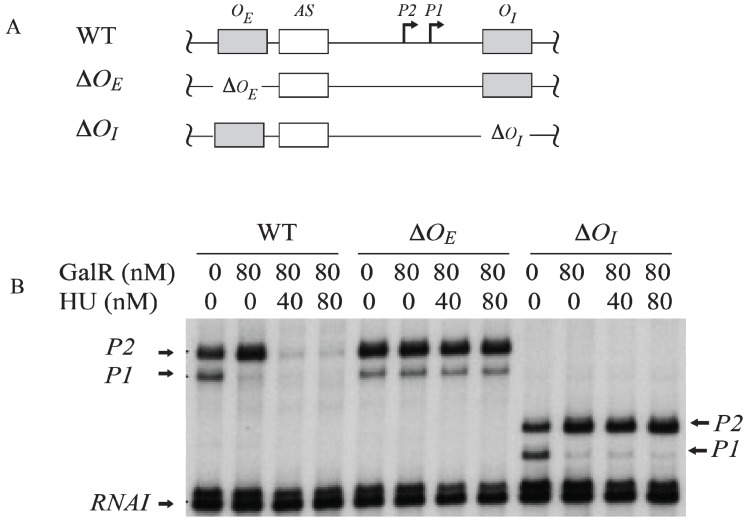
Role of the operators in the transcription regulation of *gal* promoters. (**A**) Templates showing Δ*O_E_* and Δ*O_I_* deletions; (**B**) mRNAs made from *P1* and *P2* on *O_E_* and *O_I_*, Δ*O_E_* and *O_I_*, and *O_E_* and Δ*O_I_* templates in the presence of GalR (80 nM) and HU (40 nM, 80 nM) (adapted from [54]).

To investigate the role of each operator in contact inhibition of *P1*, and contact activation of *P2*, *O_E_* or *O_I_* was subjected to mutational analysis (Figure 6A). The result shows that the GalR-*O_E_* complex formation is sufficient for the repression of *P1* (Figure 6B) [53,54,55,56]. When *O_E_* was deleted, there was no inhibition of *P1*, no activation of *P2*. When *O_I_* was deleted, GalR-*O_E_* complex still repressed *P1* and activated *P2*. When *O_I_* was deleted, the length of the transcripts from *P1* and *P2* was reduced by 16-nt since *O_I_* is located downstream of both promoters. There is no change in the length of the transcripts from *P1* and *P2* when *O_E_* was deleted because the *tsps* of the promoters are located downstream of the *O_E_*.

The operon consists of two GalR binding sites (16-bp operators), *O_E_* (external operator, located at position −60.5) and *O_I_* (internal operator within *galE*, located at +53.5) [2,4,57]. The *galE* gene starts at an ATG (methionine code) located at position starting at +27. The operators are located 113-bp (~11 DNA helical turns) from each other (center to center distance) (Figure 1). GalR binds to each operator as a dimer.

**Figure 7:**
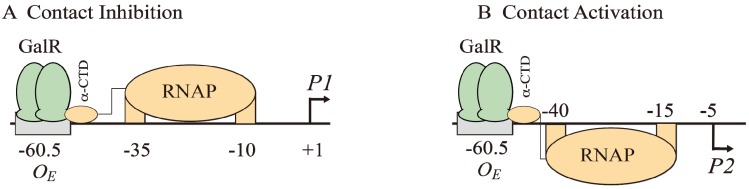
Models of GalR-RNAP contacts. (**A**) Contact inhibition of *P1*: GalR (light green) at *O_E_* is interacting with the α-CTD of RNAP (brown) at the −10 and −35 elements of *P1*; (**B**) Contact activation of *P2* with the α-CTD of RNAP at the −10 and −35 elements of *P2*.

The binding of GalR to *O_E_* represses *P1* and activates *P2* (Figure 6B and Figure 7A). GalR bound to *O_E_* is located on the same DNA face as RNAP bound to *P1* (Figure 7A), but on the opposite DNA face as RNAP bound to *P2* (Figure 7B). GalR represses *P1* by inhibiting the rate determining open complex formation through RNAP contacts [58]. This mode of repression is termed “contact inhibition” (Figure 7A). While *P1* is repressed by GalR-*O_E_* complex, *P2* is activated by GalR-*O_E_* complex by a direct contact between GalR and RNAP, “contact activation” (Figure 6B and Figure 7B). GalR-*O_E_* enhances open complex formation at *P2* presumably in the same way CCC does in *P1* [12,53,59]. In *P1*, GalR energetically traps RNAP at an intermediary complex [53]. GalR mutants (*nc*, for negative control) that bind to *O_E_* and do not repress *P1* but represses *P2* have been isolated. These mutations presumably define the contact points of GalR to which RNA polymerase binds while occupying *P1* and need to be characterized further [60]. The contact points of RNAP for GalR are unknown.

## 8. Roadblock of RNAP by GalR-*O_I_* Complex

**Figure 8:**
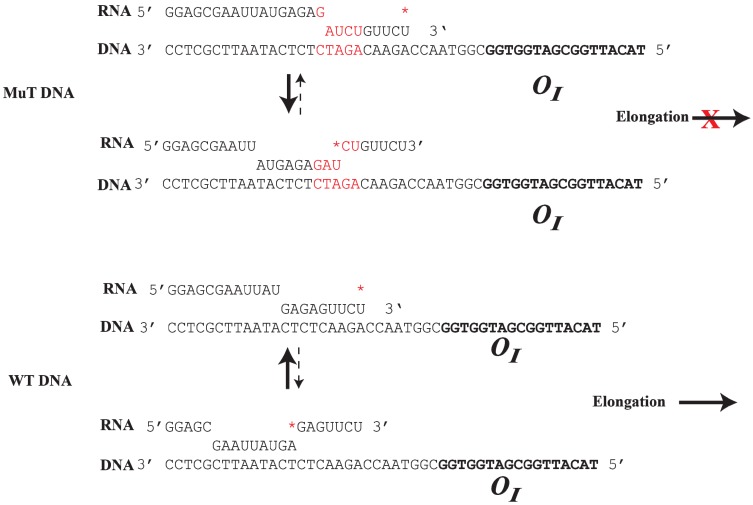
Role of DNA sequence in RNAP elongation or backtracking. (**Top**): The mutant DNA contains an insertion of 5-bp (GATCT, red color), which creates a weak RNA:DNA hybrid at the 3' end of the RNA. RNAP prefers to backtrack to a more stable RNA:DNA hybrid from a weak hydrid, preventing elongation; (**Bottom**): In WTDNA, a strong 9-bp RNA:DNA hybrid is formed at the *gal* pause site upstream from *O_I_*. RNAP prefers to elongate instead of backtracking by 7-bp to a weak RNA:DNA hybrid. The * indicates the 3' end of the RNA (reproduced with permission from Elsevier [61]).

The *O_I_* operator is located at position +53.5, which is in the path of elongating RNAP complex transcribing from *P1* and *P2*. The question is whether GalR-*O_I_* complex can inhibit or block RNAP elongation [61]. Unexpectedly, transcription from the *gal* promoters under *in vitro* conditions overrides the expected physical block created by the presence of the GalR bound to *O_I_* (Figure 8). It has been shown that although a stretch of pyrimidine residues (UUCU) in the RNA/DNA hybrid located immediately upstream of *O_I_* weakens the RNA/DNA hybrid and favors RNA polymerase pausing and backtracking after encountering the roadblock, a stretch of purines (GAGAG) in the RNA present immediately upstream of the pause sequence in the hybrid acts as an anti-pause element by stabilizing the RNA/DNA duplex and preventing further backtracking. This facilitates forward translocation of RNAP, including overriding of the DNA-bound GalR barrier at *O_I_* [61]. Consequently, when the GAGAG sequence is separated from the pyrimidine sequence by a 5-bp DNA insertion, RNAP backtracking is favored from a weak hybrid to a more stable hybrid (Figure 8). The roadblock of RNAP by GalR-*O_I_* complex in the template with the 5-bp insertion was rescued by the transcription elongation factor, GreB, but not GreA. GreB and GreA cleave backtracked RNA in the catalytic center of RNAP to create a new 3'-end of the RNA, which can then be elongated [62,63,64,65]. As expected, the roadblock is also rescued by d-galactose, which dissembles the GalR-*O_I_* complex, allowing RNAP to continue transcription [61]. The ability of a native DNA sequence to override roadblocks in transcription elongation in the *gal* operon uncovers a previously unknown way of regulating transcription.

## 9. DNA Looping

**Figure 9:**
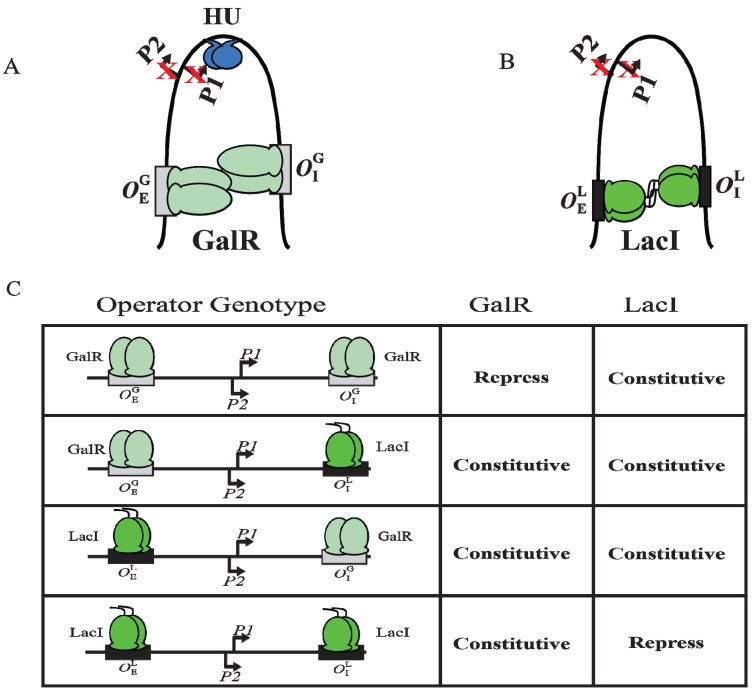
DNA looping by GalR and LacI. (**A**) Repressome formation by GalR-*O_E_* and GalR-*O_I_* interactions with HU (blue) and supercoiled DNA. DNA looping repressed both *P1* and *P2*; (**B**) DNA looping by LacI binding to *lac* operators; (**C**) The *in vivo* level of galactokinase, a product of the *gal* operon is reported as repressed or constitutive in the presence of GalR and LacI on $O_{E}^{G}-O_{I}^{G}$, $O_{E}^{G}-O_{I}^{L}$, $O_{E}^{L}-O_{I}^{G}$ and $O_{E}^{L}-O_{I}^{L}$ templates. GalR is in light green and LacI is in dark green. The *gal* operators are in grey rectangular boxes, while the *lac* operators are in black rectangular boxes (adapted from [66]).

Although GalR binding to a specific operator (*O_E_* or *O_I_*) has different regulatory outcomes, simultaneous binding of GalR to both operators (in the presence of HU; see later) represses both *P1* and *P2* (Figure 6B). It was proposed that GalR bound to the distally located operators interact with each other forming a loop of the intervening DNA that contains the promoters (Figure 9A). To test this model, a set of bipartite operators was constructed by converting *gal* operators to *lac* operators in various combinations ($O_{E}^{G}-O_{I}^{G}$, $O_{E}^{G}-O_{I}^{L}$, $O_{E}^{L}-O_{I}^{G}$, $O_{E}^{L}-O_{I}^{L}$) and *gal* repression was studied *in vivo* [66]. GalR and LacI are part of the GalR-LacI family, in which members show 60% homology in sequence [67]. Simultaneous repression of both promoters occurred only with homologous operators ($O_{E}^{G}-O_{I}^{G}$ or $O_{E}^{L}-O_{I}^{L}$) in the presence of the cognate repressor (Figure 9A–C) [66]. GalR does not recognize *lac* operators and LacI does not recognize *gal* operators. These results suggest that the occupation of both operators by heterologous proteins was not sufficient for complete repression of the promoters. It was also inferred that protein-protein interactions occur between homologous proteins bound to cognate operators to form DNA loop and being about repression.

## 10. Mechanism of Repression by DNA Looping

Although an interaction between GalR-*O_E_* and GalR-*O_I_* complexes to generate a DNA loop was predicted from the *in vivo* results, *in vitro* experiments could not demonstrate DNA looping in *gal* DNA in the presence of GalR [68]. *In vitro,* DNA looping additionally needs the presence of the histone-like protein HU and supercoiled DNA (see below). HU assists the GalR-*O_E_* complex and the GalR-*O_I_* complex in stabilizing a higher-order complex structure, resulting in a DNA loop (see below). The histone binding site (*hbs*) is located in the apex of the loop where the DNA is bent by HU binding, forming the higher-order structure and repressing both *P2* and *P1* (Figure 6B) [68]. The higher-order structure is termed “repressosome” (Figure 9A). *P2* is repressed only by the repressosome. Incidentally, repression of both *P1* and *P2* at the same time by GalR-HU mediated DNA looping overrides the GalR-*O_E_* and CCC-AS mediated DNA looping differential regulation of *P1* and *P2.* Mechanistically, synergistic binding of GalR to distal sites forms 113 bp DNA loop which is a topologically closed domain containing the two promoters [56]. A closed DNA loop of 11 helical turns, which is in-flexible to torsional changes, disables the promoters either by resisting DNA unwinding needed for open complex formation or by impeding the processive DNA contacts by an RNA polymerase in flux during transcription initiation. Interaction between two proteins bound to different sites on DNA modulating the activity of the intervening segment toward other proteins by allostery may be a common mechanism of regulation in DNA-multiprotein complexes.

As mentioned the *P1* promoter of *gal* contains only ex-10 and −10 DNA elements and no −35 element. Thus, recognition of *P1* does not require specific contacts between RNA polymerase and its −35 element region. To investigate whether specific recognition of the −35 element would affect the regulation of *P1* by GalR, variants of *P1* in which the −35 element was restored were constructed and their regulation by DNA looping were studied by *in vitro* transcription assays [69]. The results showed that the GalR-mediated DNA loop is less efficient in repressing P1 transcription when RNA polymerase binds to the −10 and −35 elements concomitantly. The most likely explanation of RNA polymerase binding to −35 element inhibiting DNA looping is that RNA polymerase binding to −35 element is known to create a bend in the DNA at an improper position inhibits DNA loop formation.

## 11. *In Vitro* Evidence of DNA Looping

**Figure 10:**
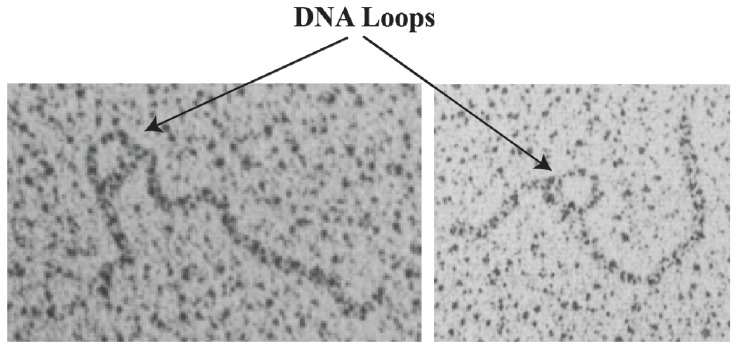
Electron micrograph of LacI-mediated DNA loop. The arrow points to the DNA loop of 95 bp in the ($O_{E}^{L}-O_{I}^{L}$) DNA. One end of the DNA is 130 bp from the loop and the other end is 380 bp (reproduced with permission from Cold Spring Harbor Laboratory Press [70]).

**Figure 11:**
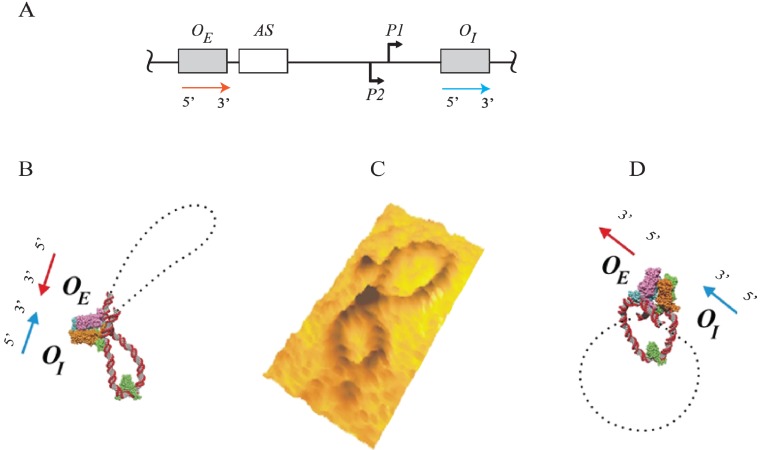
Atomic force microscopy of GalR-HU mediated DNA loops. (**A**) Sketch of the *gal* DNA with a red arrow in the direction of 5' to 3' for *O_E_* and with a blue arrow in the direction of 5' to 3' for *O_I_*; (**B**) Model of antiparallel loop with the 3' of *O_E_* and *O_I_* facing each other in a head-to-head arrangement; (**C**) AFM results showing an antiparallel loop as predicted in model (B); (**D**) Model of parallel loop with the 3' of *O_E_* and *O_I_* facing opposite direction in a tail-to-head arrangement (reproduced with permission from Elsevier [72]).

DNA looping of LacI binding to two operators ($O_{E}^{L}-O_{I}^{L}$) is supported by direct visualization using of electron micrographs (EM), showing a loop size of 95-bp with two arms in long linear DNA (Figure 10) [70]. A dimeric LacI mutant that is unable to form tetramers failed to form DNA loops. GalR-HU-DNA mediated repressosome (DNA loop) was visualized by atomic force microscopy (AFM) on DNA minicircles of 688-bp with a figure-of-eight structure containing two loops: a 113-bp loop and a large loop of 555-bp as expected [71]. However, the geometry of the loop (antiparallel or parallel) is not distinguishable by this AFM image because of the positions of the two operators in the 688-bp plasmid. To address the geometry of the loop, minicircles of 599-bp containing an additional 84-bp insertion between *O_E_* and *O_I_* is used to show a small loop size of 197-bp and a larger loop of 402-bp with GalR and HU (Figure 11) [72]. The geometry of the DNA loop (Figure 11C) is antiparallel (Figure 11B) and not parallel (Figure 11D). AFM study with LacI and *lac* operators containing the same size DNA (*lac* operators replaced the *gal* operators) also reveals an antiparallel loop [72].

## 12. Helical Arrangement of Operators

**Figure 12:**
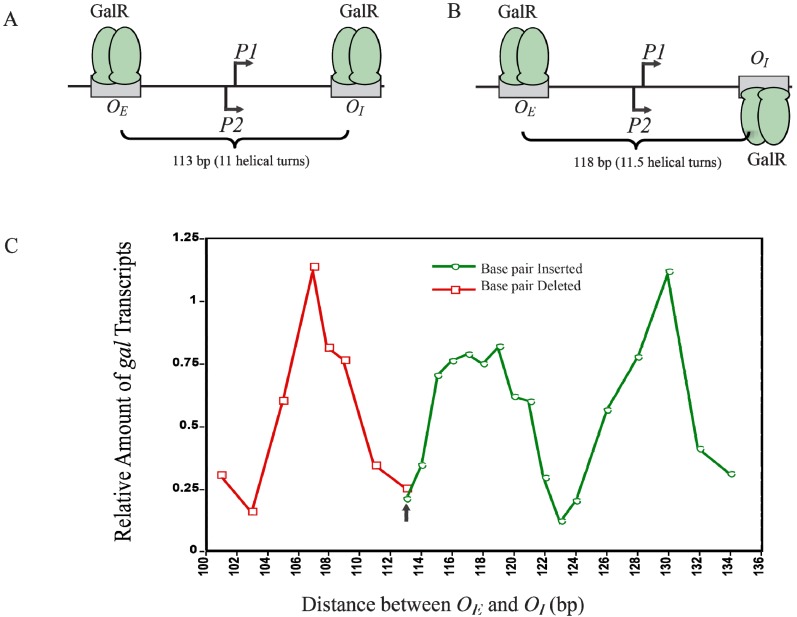
Looping-mediated repression of *gal* transcription is dependent on the helical distance between operators. (**A**) GalR-*O_E_* and GalR-*O_I_* are located on the same face of the DNA at a distance of 113-bp (11 helical turns); (**B**) GalR-*O_E_* and GalR-*O_I_* are located on the opposite face of DNA at a distance of 118-bp; (**C**) Relative amount of transcription *vs.* distance between the two operators in base pair in the presence of GalR and HU. The results of deleted base pairs (12 bp) are in red and the results of inserted base pairs are in green. The arrow indicates the WT distance between operators (adapted from [73]).

The centers of the operators *O_E_* and *O_I_* are separated by 11 DNA helical turns, thus making the location of two operator-bound GalR on the same face of DNA. This arrangement energetically allows an interaction between two DNA bound GalR dimers, and consequent DNA looping and transcription repression, (Figure 12A). To investigate the dependence of transcription repression on the relative helical turns of the location of *O_E_* and *O_I_*, the helical arrangement of *O_E_* and *O_I_* was changed by either deleting 2- to 12-bp within positions −50 to −38 to decrease the number of helical turns or by inserting 1- to 21-bp between positions +32 and +33 to increase the helical turns [73]. Figure 12C shows that the optimal repression of *gal* RNA synthesis is achieved at a net distance of 103-, 113-, 123-, and 133-bp, corresponding to 10, 11, 12 and 13 full helical turns, respectively. However, when a 5-bp segment was deleted (108-bp distance) or 5- and 15-bp segments were inserted (118- and 128-bp distance respectively), *gal* repression was lifted as judged by the high expression of RNAs even in the presence of GalR, HU and supercoiled DNA presumably making the GalR-*O_E_* and GalR-*O_I_* complexes now located on the opposite face of the DNA more difficult to make GalR-GalR contact for DNA looping (Figure 12B). Moreover, the loop size between *O_E_* and *O_I_* can be increased up to a total of 19 helical turns to maintain loping-mediated repression [72]. Above 19 helical turns, the repression was reduced, perhaps because a bound RNAP is able to overcome DNA torsional stress and form open complex.

## 13. Role of HU in DNA Looping

**Figure 13:**
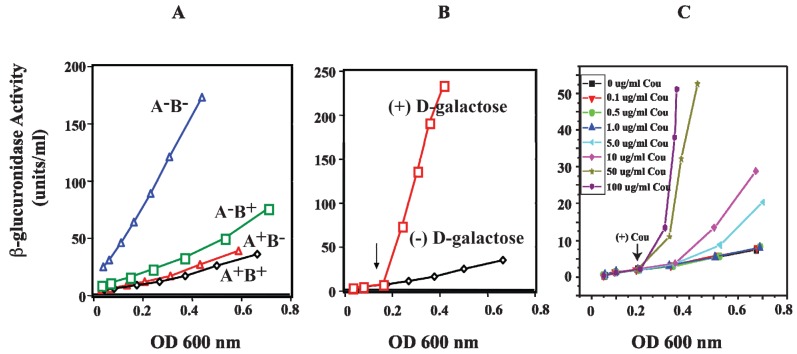
Effect of HU and d-galactose on *P2 in vivo*. (**A**) β-glucuronidase activity as a reporter of the *P2* promoter in WT (*hup*A^+^B^+^) and mutants (*hup*A^+^B^−^, *hup*A^−^B^+^ and *hup*A^−^B^−^) strains; (**B**) β-glucuronidase activity from *P2* in WT (*hup*A^+^B^+^) strain in the absence and presence of d-galactose; (**C**) β-glucuronidase activity from *P2* in WT (*hup*A^+^B^+^) strain in the presence of various coumermycin concentrations. The arrow indicates where d-galactose or coumermycin was added. In each panel, the x-axis shows cell OD (reproduced with permission from John Wiley and Sons Ltd. (Hoboken, NJ, USA) [78]).

HU is a small basic protein consisting of a heterodimer of α and β subunits, and binds to a 9-bp DNA [74,75]. The *hup*A gene codes for Huα (~9 kDa) and the *hup*B gene codes for Huβ (~9 kDa) [76,77]. The involvement of HU in DNA looping and the repression of the *gal* operon were investigated *in vivo* by monitoring the activity of β-glucuronidase from *gus*A fused to the *P2* promoter. The *hup*A and *hup*B genes were deleted (Δ*hupA*::cm^R^ and Δ*hupB*::km^R^) to generate *hup*A^+^B^−^, *hup*A^−^B^+^, and *hup*A^−^B^−^ strains [78]. In wild-type strain (*hup*A^+^B^+^), the β-glucuronidase activity of *P2* was repressed when both genes are present (Figure 13A). In the presence of the *hup*A gene (*hup*A^+^B^−^), *P2* is strongly repressed as in wild-type cells, suggesting that *hup*A is sufficient to achieve complete repression of *P2* by DNA looping. When *hup*A is inactivated (*hup*A^−^B^+^), the repression of *P2* is slightly weaker than that for *hup*A^+^B^+^ and *hup*A^+^B^−^. The derepression of β-glucuronidase activity of *P2* was completely constitutive only in the *hup*A^−^B^−^ strain. The *P2* activity of *hup*A^−^B^−^ is comparable to that of *P2* activity in the presence of d-galactose, an inducer of the *gal* operon (Figure 13B) [78].

## 14. DNA Supercoiling

DNA loping by GalR and HU occurs only with supercoiled DNA as was observed by *in vitro* transcription of *P2*. *P2* repression was totally dependent upon with supercoiled DNA template [78]. Moreover DNA looping mediated repression *in vivo* requires supercoiled chromosome [71]. Coumermycin, a DNA gyrase inhibitor, also derepressed the *P2* promoter as expected when it was added to cells in the absence of d-galactose (Figure 13C).

## 15. Piggybacking HU

**Figure 14:**
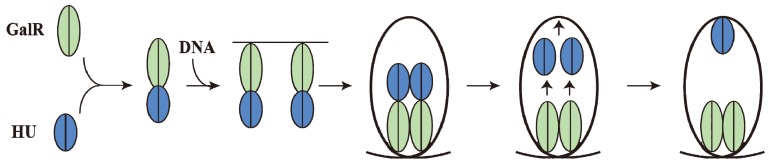
Pathway of Repressosome formation. GalR (light green) and HU (blue) first bind together, then bind to the DNA, resulting in DNA loop formation with HU dissociating from GalR and binding to the apex of the DNA stabilizing the loop involving GalR-GalR interactions (adapted from [80]).

GalR mediated DNA looping requires binding of HU to an architecturally critical position on DNA (*hbs*) to facilitate the GalR-GalR interaction. It has been shown that GalR piggybacks HU to the critical position on the DNA through a specific GalR-HU interaction [79]. The thermodynamic parameters of some of the required interactions, GalR-*O_E_*, GalR-GalR, HU-GalR, and HU-GalR-*O_E_*, were studied by analytical ultracentrifugation, fluorescence anisotropy, and fluorescence resonance energy transfer [80]. The physiological significance of several of these interactions was confirmed by the finding that a mutant HU, which is unable to help looping *in vivo* and *in vitro*, failed to show the HU-GalR interaction. The results helped to construct a pathway of DNA looping (Figure 14). Structure-based genetic analysis indicated that the two DNA-bound GalR dimers interact directly and form a stacked tetramer in assembling a transient loop [81]. The loop is stabilized by HU leaving GalR and binding to the architecturally critical position on the DNA. The GalR-HU contact is likely transient and absent in the final loop structure. A sequence-independent DNA-binding protein being recruited to an architectural site on DNA through a specific association with a regulatory protein may be a common mode for assembly of complex nucleoprotein structures [80].

## 16. DNA Loop Trajectory

**Figure 15:**
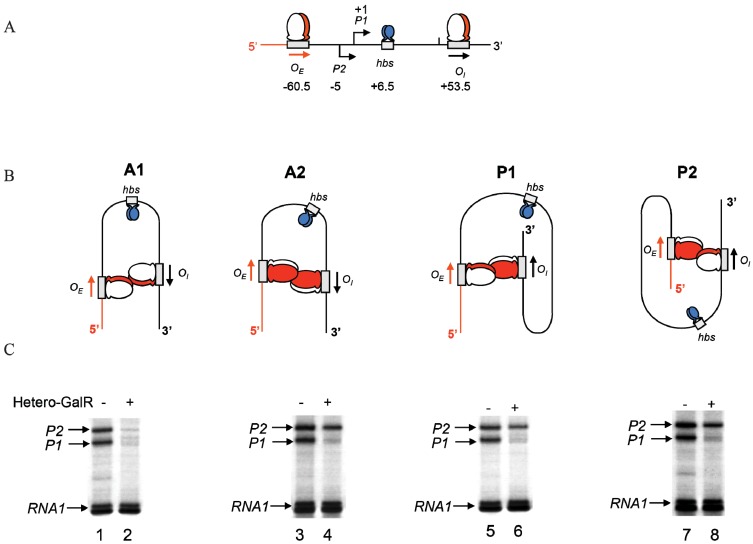
Trajectory of DNA loops formed by GalR and HU; (**A**) The *gal* regulatory region containing promoters (*P1* and *P2*), operators (*O_E_* and *O_I_*), HU binding site (*hbs*). The arrows at *O_E_* and *O_I_* are shown in the direction 5' to 3'; (**B**) Two trajectories of DNA loops, “antiparallel” (A), and “parallel” (P); (**C**) mRNAs made from *P1* and *P2* in antiparallel or parallel configurations (reproduced with permission from Cold Spring Harbor Laboratory Press, [5]).

In the scheme of DNA looping as shown in Figure 14, the alignment of the operators in the DNA loop could be in either parallel (P) or antiparallel (A) mode (Figure 15). Feasibilities of these trajectories were tested by *in vitro* transcription repression assays, first by isolating GalR mutants with altered operator specificity and then by constructing proper operator sequences to allow formation of mutant GalR heterodimers bound to specific hybrid operators in such a way as to give rise to only one of the two putative trajectories (parallel (P) or antiparallel (A)) [5]. A1 loop is formed when the 3'-end of *O_E_* is facing the 3'-end of *O_I_* in a head-to-head (→ ←) orientation. The A2 loop is formed when the 3'-end of *O_E_* is facing away from the 3'-end of *O_I_* in a tail-to-tail (← →) orientation. In P1, the 3'-end of *O_E_* is located in the same direction as the 3'-end of *O_I_* in a head-to-tail (← ←) configuration, while in P2, the 3'-end of *O_E_* is located in the opposite direction as the 3'-end of *O_I_* in a tail-to-head (→ →) configuration. Results show that *O_E_* and *O_I_* adopt a mutual antiparallel orientation in an under-twisted DNA loop, consistent with the energetically optimal structural model. In this structure the center of the HU-binding site is located at the apex of the DNA loop (Figure 15).

## 17. Single Molecule Evidence of DNA Looping

**Figure 16:**
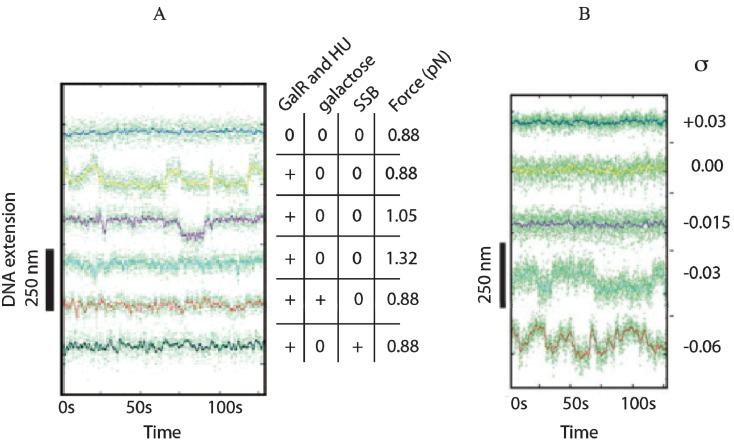
Single molecules of DNA looping. (**A**) Traces of DNA in the presence of GalR, HU, d-galactose or single-stranded DNA (SSB) with DNA supercoiling (σ = −0.03) and magnetic forces of 0.88, 1.05 and 1.32 pN; (**B**) DNA looping *vs.* superhical density (σ) (reproduced with permission from the National Academy of Sciences, USA, [83]).

Single DNA molecule experiment was first used to demonstrate looping in the *lac* operon with a linear DNA [82]. The same principle was employed to demonstrate DNA looping in *gal* in which the extra factor HU and supercoiling of the DNA were needed [83]. Single DNA molecules each containing two operator sequences 113 bp apart, with one end tethered to a magnetic bead and the other to a surface can be twisted to mimic DNA superhelicity by using small magnets placed above the sample and the end-to-end distance measured. Under such conditions DNA loop formation by GalR and HU reduced the bead-to-surface distance by an expected amount. GalR/HU-mediated DNA looping was directly detected and characterized for its kinetics, thermodynamics, and supercoiling dependence. Transitions in DNA length between unlooped state and looped state were observed in the presence of GalR and HU (Figure 16A). There was no transition in the absence of either GalR or HU. The optimal super helical density (σ) for looping was −0.03. Looping was not observed with untwisted (relaxed) DNA making negative supercoiling an essential element for looping in this system unlike loop formation in *lac* (Figure 16B) [82]. These experiments also confirmed that DNA looping in *gal* occurs with an antiparallel DNA trajectory of the two operator [84].

## 18. GalR-GalR Interface for DNA Looping

How does GalR*-O_E_* complex interact with GalR*-O_I_* complex to bring about tetramerization and DNA looping? This question was addressed by isolation and characterization of single amino acid *gal*R mutants, which bind to DNA (*P1* repression proficient) but does not form DNA loop (*P2* repression deficient) [85]. A reporter gene of *O_E_* *P1*^+^*P2*^−^~*lac*Z was used to monitor *P1* repression and an *O_E_* *P1*^−^*P2*^+^*O_I_* ~*gus*A fusion was used to screen for *P2* repression. Such GalR mutants (defective in GalR-GalR interactions, and thus DNA looping but retains DNA binding to *O_E_*), R325H, D258N and E230K, were located on a surface of a model structure of GalR dimer structure. The area can act as interface between two GalR dimers [85]. *In vitro* studies confirmed that the interface mutants, R325H, D258N and E230K, do not repress *P2* but repress *P1*.

## 19. Induction of *Gal* Operon by d-Galactose

**Figure 17:**
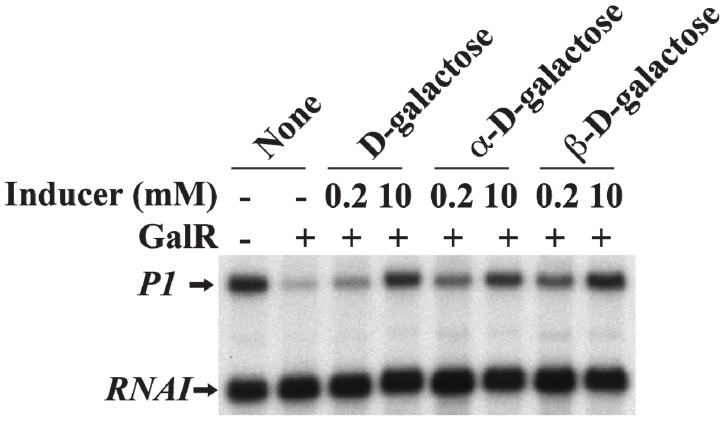
Derepression of *P1* by d-galactose. mRNAs made from *P1* in the presence of GalR (80 nM) and d-galactose, α-d-galactose or β-d-galactose 0.2 and 10 nM) (adapted from [86]).

d-Galactose, an inducer of GalR, acts by inactivating GalR. d-galactose binding to GalR results in an allosteric change in the protein, which cannot contact RNAP or bind to DNA anymore. This neutralizes any regulatory effect of GalR. d-galactose is a mixture of both α-anomer and β-anomer [86]. Purified α-anomer and β-anomer were used to investigate whether the α-anomer or β-anomer or both can inactivate GalR for *P1* transcription from the repressed state of the promoter *in vitro*. The result showed that both α-anomer and β-anomer act as inducer by lifting the repression of *P1* transcription without DNA looping (Figure 17).

How does d-galactose disrupt the repressosome structure? In case of DNA looping mediated repression of *P2*, the presence of d-galactose first breaks up GalR-GalR tetramers into individual operator-bound dimers in which state *P1* is still largely repressed and *P2* is derepressed [87]. Next, d-galactose helps to dissociate GalR from GalR-*O_I_* complex, and finally, GalR dissociates from GalR-*O_E_* complex to disassemble the remaining complex. This also confirms that GalR-*O_E_* complex is more stable than GalR-*O_I_* complex [87].

## 20. Conclusions

The *gal* operon of *E. coli* plays an important role in cellular metabolism by encoding enzymes that catalyze conversion of d-galactose to energy sources as well as to anabolic substrates. The operon is transcribed from two overlapping promoters, *P1* and *P2*. The importance of individual base pairs at various positions in the −49 to +1 segment in the *gal* promoters for transcription and its regulation were discerned by substitutions, deletions or insertions of base pairs. First, a 12-bp distance from the master base pair (−11) is a determinant of transcription start point (+1), which is preferably a purine. Second, two of the standard RNAP recognition elements, ex-10 and −10, are responsible for determining the strength of the two *gal* promoters. Genetic analysis of the two overlapping promoters also identified that the DNA sequence of the segment −20 to −16, which is outside the boundaries of the previously defined promoter elements, contribute to promoter strength in both *P1* and *P2*.

Regulatory proteins, CCC and GalR, regulate the two promoters coordinately and differentially depending on the cellular conditions. (i) CCC binds to the *AS* element at position −41.5 and activates *P1* and represses *P2* both at the step of open complex formation; (ii) GalR acts by binding to two operators, *O_E_* and *O_I_*. The GalR-*O_E_* complex inhibits *P1* and stimulates *P2* by contacting the αCTD of RNAP by preventing open complex formation at *P1* and enhancing open complex formation at *P2*; (iii) Interestingly, the GalR-*O_I_* complex does not create a road-block to any elongating RNAP from *P1* and *P2* because of the presence of anti-pause sequence in the immediate upstream area that occludes pause; (iv) In the presence of the histone-like protein, HU, and supercoiled DNA as template, interactions of the two operator-bound GalR, results in the formation of a DNA loop (repressosome). The trajectory of DNA in the loop is antiparallel, as revealed by biochemical experiments and AFM observations. Genetic analysis of GalR identified the protein interface between GalR-*O_E_* and GalR-*O_I_* at the looped state. Looping needs the binding of HU to the apex of the looped DNA that stabilizes the repressosome complex. GalR interacts with HU and piggybacks the latter to its binding site. In the final structure, there is no GalR-HU contact. The helical arrangement between GalR-*O_E_* and GalR-*O_I_* is important for facilitating DNA looping. When they are located on the same face of the DNA looping is favorable; when not on the same face, energetics prevents DNA looping.
